# Suborganellar resolution imaging for the localisation of human glycosylation enzymes in tobacco Golgi bodies

**DOI:** 10.1111/jmi.13311

**Published:** 2024-04-30

**Authors:** Alastair J. McGinness, Susan A. Brooks, Richard Strasser, Jennifer Schoberer, Verena Kriechbaumer

**Affiliations:** ^1^ Department of Biological and Medical Sciences Oxford Brookes University Oxford UK; ^2^ Department of Applied Genetics and Cell Biology University of Natural Resources and Life Sciences Vienna Austria; ^3^ Centre for Bioimaging Oxford Brookes University Oxford UK

**Keywords:** cytoplasmic‐transmembrane‐stem regions, glycosylation, Golgi cisternae, human glycosylation enzymes

## Abstract

Plant cells are a capable system for producing economically and therapeutically important proteins for a variety of applications, and are considered a safer production system than some existing hosts such as bacteria or yeasts. However, plants do not perform protein modifications in the same manner as mammalian cells do. This can impact on protein functionality for plant‐produced human therapeutics. This obstacle can be overcome by creating a plant‐based system capable of ‘humanising’ proteins of interest resulting in a glycosylation profile of synthetic plant‐produced proteins as it would occur in mammalian systems.

For this, the human glycosylation enzymes (HuGEs) involved in N‐linked glycosylation N‐acetylglucosaminyltransferase IV and V (GNTIV and GNTV), β‐1,4‐galactosyltransferase (B4GALT1), and α‐2,6‐sialyltransferase (ST6GAL) were expressed in plant cells. For these enzymes to carry out the stepwise glycosylation functions, they need to localise to late Golgi body cisternae. This was achieved by a protein targeting strategy of replacing the mammalian Golgi targeting domains (Cytoplasmic‐Transmembrane‐Stem (CTS) regions) with plant‐specific ones. Using high‐resolution and dynamic confocal microscopy, we show that GNTIV and GNTV were successfully targeted to the medial‐Golgi cisternae while ST6GAL and B4GALT1 were targeted to *trans‐*Golgi cisternae.

Plant cells are a promising system to produce human therapeutics for example proteins used in enzyme replacement therapies. Plants can provide safer and cheaper alternatives to existing expression systems such as mammalian cell culture, bacteria or yeast. An important factor for the functionality of therapeutic proteins though are protein modifications specific to human cells. However, plants do not perform protein modifications in the same manner as human cells do. Therefore, plant cells need to be genetically modified to mimic human protein modifications patterns. The modification of importance here, is called N‐linked glycosylation and adds specific sugar molecules onto the proteins.

Here we show the expression of four human glycosylation enzymes, which are required for N‐linked glycosylation, in plant cells.

In addition, as these protein modifications are carried out in cells resembling a factory production line, it is important that the human glycosylation enzymes be placed in the correct cellular compartments and in the correct order. This is carried out in Golgi bodies. Golgi bodies are composed of several defined stacks termed *cis*‐, medial and *trans*‐Golgi body stacks. For correct protein function, two of these human glycosylation enzymes need to be placed in the medial‐Golgi attacks and the other two in the *trans*‐Golgi stacks. Using high‐resolution laser microscopy in live plant cells, we show here that the human glycosylation enzymes are sent within the cells to the correct Golgi body stacks. These are first steps to modify plant cells in order to produce human therapeutics.

## 1 INTRODUCTION

### 1.1 Production of recombinant proteins in plant systems

Plant systems for the production of recombinant protein are a fast‐growing industry. Major benefits of plant‐based systems for recombinant protein production are low‐cost, sustainable scalability, reliable stable or transient expression and flexibility of genetic modification.^1^ The innate tolerance of mammals to plant proteins, through everyday contact and diet, makes plant‐produced therapeutics highly desirable when compared to those produced in vectors such as bacteria, because of their lack of a substantial immunogenic profile.^1^ Similarly, yeast and bacterial systems can generate high risk immunogenic contaminants, that is, bacterial lipopolysaccharides and terminally mannosylated glycan structures; these are a risk factor for therapeutic applications as they can trigger detrimental immune responses in patients.^2^

Plant‐based platforms begin to challenge existing, well‐developed mammalian and bacterial systems, as industry begins to see the economic and biological advantages of biopharming.^2^ Therapeutic potential for plant‐manufactured proteins has real‐world demonstrations of effectiveness, including pandemic response capability of tobacco‐manufactured therapeutics for the 2014 Ebola outbreak,^3^ to the first plant‐manufactured enzyme replacement therapy of recombinant *Taliglucerase alfa* therapy, utilised for Gaucher's disease and produced in carrot cell culture.^4^ Proteins of commercial interest, such as antibodies and enzymes, frequently require extensive and specific posttranslational modification in order to be functional or therapeutically effective. Lysosomal acid lipase, a recombinant protein for enzyme replacement therapy in lipid storage disorders, and immunoglobulins (IgA, IgM, IgE and IgG) are evidence of the requirement for correct protein synthesis as well as posttranslational modification, and have multiple N‐linked glycosylation sites. Plant systems can perform important posttranslational modifications such as N‐linked glycosylation but do not generate the same late Golgi glycan modifications that occur in mammalian N‐glycosylation, lacking key mammal‐specific motifs.

### 1.2 N‐linked glycosylation for functional therapeutic proteins

**FIGURE 1:**
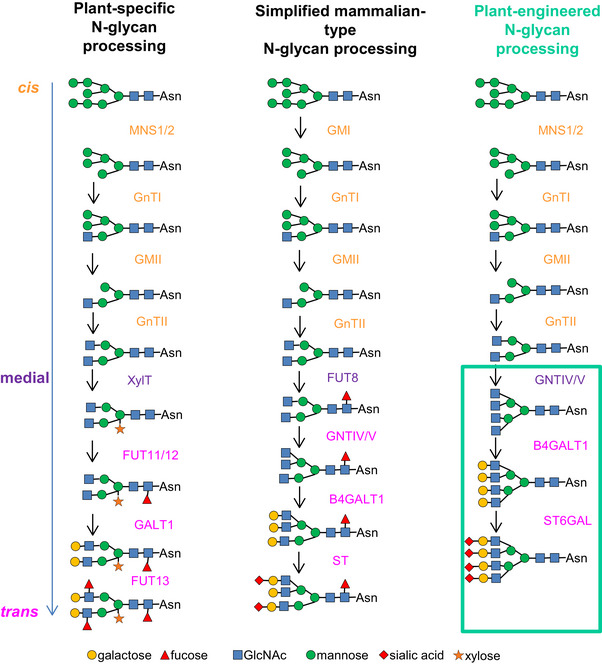
Comparison of plant and mammalian N‐linked glycosylation and modifications required for humanising plant N‐linked glycosylation. Required modifications to the plant system in terms of insertion of mammalian enzymes and their resultant glycan structures are highlighted in the green box on the right hand side. Enzymes are colour‐coded for localisation in *cis*‐Golgi cisternae (orange), medial‐Golgi cisternae (purple) and *trans*‐Golgi cisternae (magenta). MNS: α‐mannosidase, GnT: N‐acetylglucosaminyltransferase, XylT: β‐1,2‐xylosyltransferase, FUT11/12: α−1,3‐fucosyltransferase, GALT1: β‐1,3‐galactosyltransferase, FUT13: α−1,4‐fucosyltransferase. Modified from Ref. (8). Symbol nomenclature for graphical representation of glycans is based on.^17^

N‐linked glycosylation refers to the stepwise enzymatic attachment of sugar moieties onto specific asparagine residues residing on synthesised protein^5^ and has a strong impact on protein localisation and function.^6^ The composition of glycosylation enzymes in plant and mammalian systems is comparable in the early stages of N‐glycan chain generation in the *cis*‐Golgi cisternae, but differs in the enzymes present in the late medial/*trans*‐Golgi ^7^, ^8^ (Figure 1). N‐acetylglucosaminyltransferases (GnTs; GnTI, GnTII, GnTIV and GnTV) use Uridine diphosphate‐*N*‐acetylglucosamine (UDP‐GlcNAc) in order to transfer N‐acetylglucosamine onto various points of the developing glycan structure, onto which further specific galactosylation is performed by B4GALT1, and final sialic acid capping of the glycan structure by ST6GAL (Figure 1).^9^ The former steps (GnTI and GnTII) are conserved between plants and mammalian cells (Figure 1), while the latter and subsequent steps responsible for the branching (formation of tri‐ and tetra‐antennary glycans) and end‐terminal sialylation (GNTIV, GNTV, B4GALT1 and ST6GAL) are not present in plants and must be introduced for sialic acid capping to occur.^9^

While native plants enzymes can functionally N‐glycosylate expressed heterologous human proteins, they do not possess the late Golgi enzymes required for the generation of a multiantennary N‐glycan chain with end‐terminal sialic acid capping, which is the required structure of mammalian N‐glycans. Adaptation of the plant machinery to produce human glycan patterns requires the addition of mammalian enzymes GNTIV and GNTV (N‐acetylglucosaminyltransferase IV and V), B4GALT1 (β‐1,4‐galactosyltransferase), and ST6GAL (α‐2,6‐sialyltransferase), and potentially downregulation of the plant enzymes XylT (β‐1,2‐xylosyltransferase), FUT11 and FUT12 (α‐1,3‐fucosyltransferases) to avoid interference with plant glycosylation patterns. The plant enzymes to be replaced or outcompeted are located in the medial*/trans*‐Golgi, hence GNTIV, GNTV, ST6GAL and B4GALT1 should also to be targeted to medial/*trans‐*Golgi cisternae for effective enzymatic function on N‐glycan intermediates^8^ as their activity is sequential and follows the activity of the enzymes in the cis‐Golgi cisternae.

In plant Golgi bodies, this glycosylation machinery differs from the mammalian equivalent, and so is incapable of producing specific mammalian glycomotifs and a mammalian‐type glycosylation profile (Figure 1).^6^, ^7^, ^8^ The differences between plant and mammalian N‐glycosylation machinery is of importance for the expression of mammalian proteins as many economically important recombinant proteins are being glycosylated.^10^ For example, absence of terminal sialic acid significantly reduces plasma half‐life in subject testing, and is essential in many drugs for optimal therapeutic potency, as absence would significantly diminish therapeutic effectiveness of treatment.^5^ For example, the absence of complex N‐glycans in an antirabies monoclonal antibody produced in plant systems reduces its efficacy by diminishing plasma half‐life.^11^ Furthermore the introduction or amplification of key mammalian glycans can significantly improve plasma half‐life for therapeutic proteins which has been shown for example in a modified erythropoietin with enhanced end‐terminal sialyation.^12^

Overall, glycol‐engineering in plants has been shown to be possible. Production of mammalian glycan motives in plants can be achieved by the targeted heterologous expression of mammalian glycosylation enzymes.^6^, ^13^ Earlier studies demonstrating that a mammalian glycosyltransferase can convert N‐glycans in plants in a similar way as in human cells^14^, ^15^ paved the way for reconstructing mammalian glycosylation pathways in plant systems. For example, the simultaneous overexpression of six mammalian glycosylation proteins in *N. benthamiana* resulted in the synthesis of the activated sialic acid and in planta protein sialylation.^16^

To humanise plant glycosylation patterns in order to produce economically and therapeutically important proteins, insertion of the human glycosylation enzymes (HuGEs) GNTIV and GNTV, B4GALT1 and ST6GAL into the late Golgi cisternae is required. Effectiveness of enzyme knock‐in strategies will be dependent on correct localisation within the plant Golgi body to the medial and *trans*‐Golgi cisternae respectively, which can be evaluated with high‐resolution confocal dynamic imaging.

### 1.3 Glycosylation enzyme distribution in plant Golgi stacks

**FIGURE 2:**
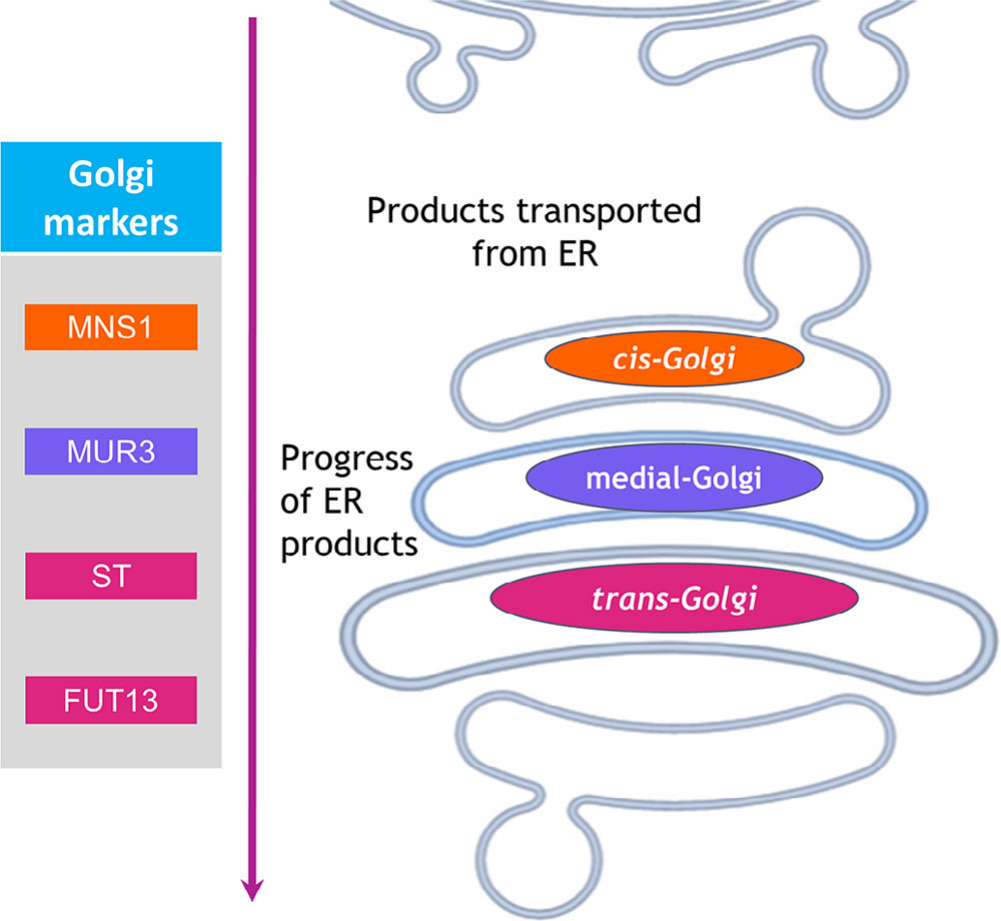
Schematic representation of suborganellar organisation of the plant Golgi body cisternae, with relevant cisternae‐specific markers colour‐coded. Orange = *cis‐*Golgi targeting (MNS1, CTS of Golgi‐α‐mannosidase I), Purple = medial‐Golgi targeting (MUR3, CTS of β‐1,2‐galactosyltransferase), Magenta = *trans‐*Golgi targeting (ST, CTS of *Rattus norvegicus* α‐2,6‐sialyl transferase; FUT13, CTS of α‐1,4‐fucosyltransferase).

N‐linked glycosylation in plants occurs within the Golgi bodies, which is a significant site of protein, lipid and polysaccharide synthesis as well as glycomodification within the plant cell, and so a focal point for production of recombinant proteins.^8^, ^18^ In contrast to the immobile, perinuclear Golgi body found in mammalian cells, Golgi bodies in higher plants are discrete stacks that can number several hundred per cell and are extremely mobile throughout the cytoplasm.^19^ The basic structure of the individual Golgi body (Figure 2) is a stack of disc‐like cisternae characterised by ordering within the stack, the *cis‐*cisternae as the entry point for cargo exiting the ER, the medial‐cisternae and the *trans‐*cisternae. From the *trans‐*cisternae the product is delivered to the Trans Golgi Network mediating intracellular delivery of ER products.^19^

Plant and human Golgi‐resident N‐glycosylation enzymes are all type II membrane proteins, each possessing at the N‐terminal region a short cytoplasmic domain, a transmembrane domain, and a stem region (CTS region). The CTS region is responsible both for orientation of the enzyme's catalytic domain in the Golgi lumen, and providing sub‐Golgi localisation, offering an explanation to the non‐uniform distribution of glycosylation enzymes in the Golgi.^20^ Existing markers (MNS1 for *cis*‐Golgi cisternae^21^, ^22^ and ST for medial/*trans*‐Golgi cisternae^23^) consist of fluorophore fusions to respective CTS domains, providing cisternae‐specific localisation as a comparison with the plant‐specific CTS domains MUR3, FUT13^24^ (Figure 2). The use of endogenous plant CTS domains can alter the localisation of human glycosylation enzymes (HuGEs), through fusion of a HuGE catalytic region with a plant CTS directed to the relevant sub‐Golgi compartment.^13^

Here we describe the transient expression and localisation of human glycosylation enzymes (HuGEs) in tobacco plants as a model system, with directed localisation to medial‐ and *trans*‐Golgi body cisternae, respectively. Suborganellar localisation to Golgi body cisternae is analysed using high‐resolution live‐cell and dynamic confocal microscopy.

## 2 RESULTS AND DISCUSSION

### 2.1 Unmodified HuGEs express weakly and do not localise specifically to the plant Golgi body

**FIGURE 3:**
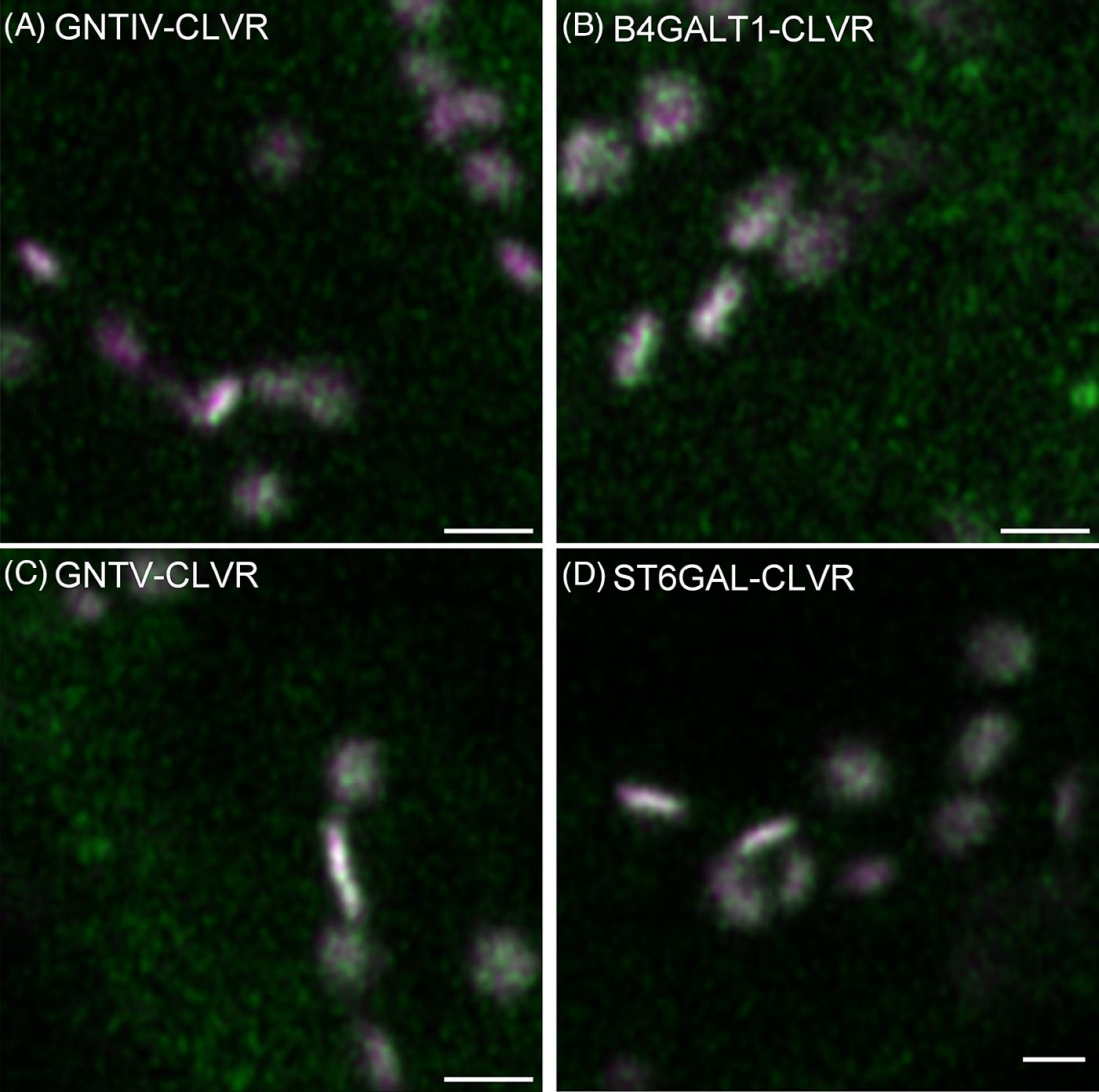
Example confocal images for unmodified HuGEs. (A) GNTIV‐CLVR, (B) B4GALT1‐CLVR, (C) GNTV‐CLVR, (D) ST6GAL‐CLVR (green) at 3 days postinfiltration alongside the *cis*‐Golgi marker MNS1‐mRFP (magenta). Representative images are shown. Size bars = 1 µm.

To investigate localisation of unmodified HuGEs featuring the mammalian signal sequences *in planta* constructs were generated for HuGEs, fused to a C‐terminal CLOVER (CLVR) fluorescent tag and expressed in *Nicotiana tabacum* (tobacco) leaf epidermal cells using agrobacterium‐mediated plant transformation. To evaluate localisation, HuGEs were co‐expressed with the *cis‐*Golgi marker MNS1‐mRFP^25^ (Figure 3). We observed low levels of expression and protein localisation did not appear specific to the plant Golgi bodies. Without Golgi‐specific localisation, HuGEs are unlikely to correctly glycosylate proteins, and so methods of targeting HuGE localisation were explored.

### 2.2 Analysis of subcellular localisation of mammalian glycosylation enzymes in tobacco leaf cells

N‐linked glycosylation requires stepwise addition of sugar moieties at each sequential Golgi body compartment. Therefore, individual glycosylation enzymes need to be located in specific Golgi cisternae to access substrate and achieve efficient glycosylation.^26^ In order to establish effective glycan processing, it was necessary to determine the sub‐Golgi localisation of HuGEs, and to target the enzymes to the late Golgi cisternae.

**FIGURE 4:**
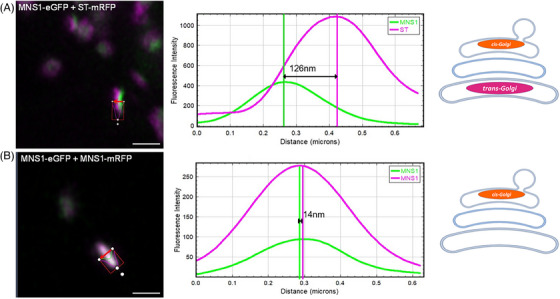
Co‐localisation of known plant Golgi cisternae markers. Line profiles for fluorescent intensity analysis across a Golgi body, with known markers (A) MNS1‐eGFP+ST‐mRFP localised to separate Golgi body cisternae to demonstrate the distance between peaks, or (B) MNS1‐eGFP+MNS1‐mRFP double markers for the same Golgi body cisterna in two colours to demonstrate overlap of expression within one Golgi body cisterna. Example confocal images and graphs are representative for the dataset. Size bars = 1 µm.

HuGEs were expressed as fluorescent protein fusions in tobacco leaf epidermal cells and their localisation to Golgi cisternae determined using statistical line profile analysis of fluorescence intensity for localisation analysis of closely associated proteins.^27^ For this approach, Golgi cisternae were imaged in side profile showing the distribution of HuGEs across the Golgi body cisternae. Analysis of *cis‐* and medial/*trans‐*Golgi markers (MNS1‐eGFP+ST‐mRFP) provided a relative baseline for the maximum distance between Golgi cisternae (Figure 4A), when compared to markers which localise to the same Golgi body cisterna (MNS1‐eGFP with MNS1‐mRFP; Figure 4B).

**FIGURE 5:**
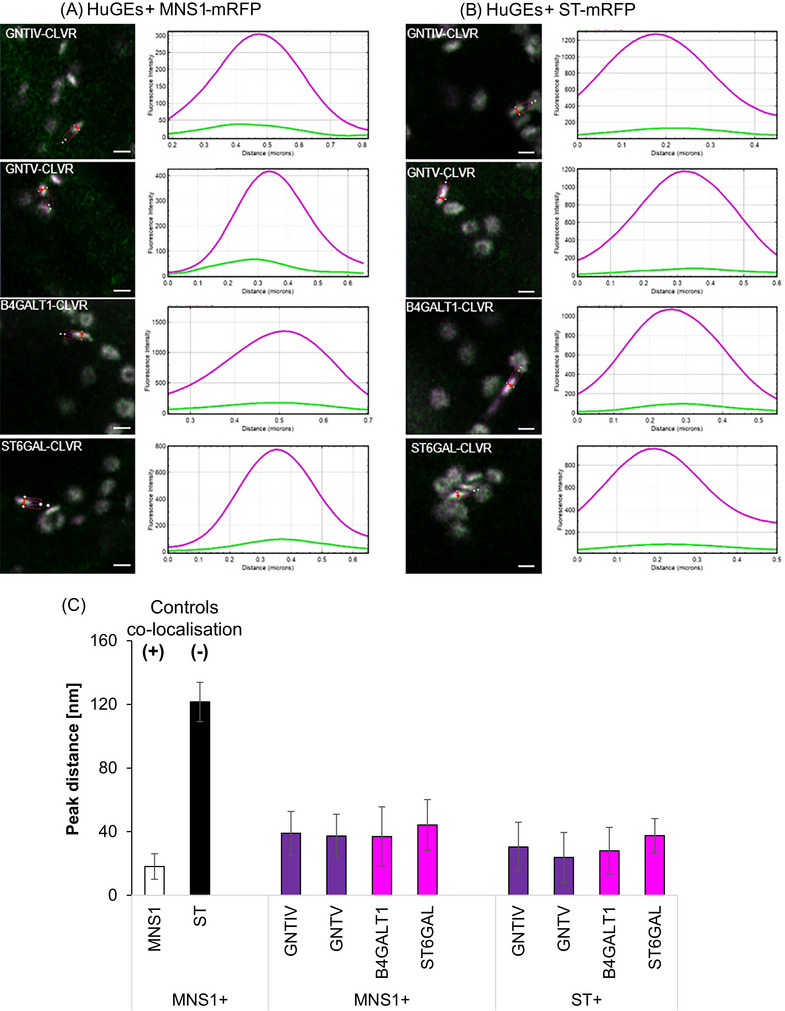
Co‐localisation line profile analysis of unmodified HuGEs. (A) Example images and analysis for statistical line profile analysis of distances between peak fluorescent intensity for GNTIV‐CLVR, GNTV‐CLVR, B4GALT1‐CLVR, ST6GAL‐CLVR (green), with *cis*‐Golgi marker MNS1 or (B) medial/*trans*‐Golgi body marker ST‐mRFP, respectively (magenta). (C) Peak distance analysis graphs showing controls for co‐localisation in the same Golgi cisternae (MNS1‐eGFP+MNS1‐mRFP, white bar) and for localisation in different Golgi cisternae (MNS1‐eGFP+ST‐mRFP, black bar) as well as comparison of the four HuGEs (medial HuGEs in purple and *trans*‐Golgi HuGEs in magenta) with the respective markers MNS1‐mRFP and ST‐mRFP. Representative images are shown. Results shown from *n* = 3 biological replicas and >12 technical repeats for each combination. Size bars = 1 µm.

HuGEs were co‐expressed with both the *cis‐*Golgi marker MNS1 and the medial/*trans*‐Golgi marker ST separately (Figure 5A) to determine their localisation within the Golgi stack. Markers for specific Golgi cisternae were used as control and a statistical analysis was carried out (Figure 5B). Co‐expression of MNS1‐eGFP with MNS1‐mRFP represented co‐localisation in the same Golgi body cisternae and showed a distance between the maximum peak intensities of 18.1 ± 8.2 nm. Co‐expression of the *cis*‐Golgi marker MNS1‐eGFP and the medial/*trans*‐Golgi marker ST‐mRFP^28^, ^29^ showed a distance between maximum peak intensities of 121.6 ± 12.4 nm, which was considered as localisation to different Golgi body cisternae.

Line profiles generated across plant Golgi bodies for all native HuGEs (Figure 5) indicated a low level of fluorescence compared to the co‐expressed marker control MNS1‐mRFP. Medial‐Golgi HuGEs GNTIV‐CLVR and GNTV‐CLVR (Figure 5) demonstrated similar distances between maximum peaks of intensity when expressed alongside MNS1‐mRFP and ST‐mRFP (ranging from 23 to 39 nm peak distance). This indicates localisation to both early and late Golgi cisternae without the required specific localisation to the late Golgi. *Trans*‐Golgi HuGEs also showed unspecific Golgi cisternae localisation; B4GALT1‐CLVR and ST6GAL‐CLVR (Figure 5) demonstrated similar distance between peaks with MNS1‐mRFP and ST‐mRFP (range 29–41 nm).

The lack of *trans*‐Golgi‐specific localisation for ST6GAL was especially surprising as the CTS regions of human ST6GAL and the marker (*Rattus norvegicus*) ST are highly conserved.^20^, ^30^ Potential explanations could be differences due to the low expression levels, an impact of the ST6GAL catalytic domain on the localisation, or the presence of some specific amino acid changes in the CTS that could affect the localisation.

With all four HuGEs showing no specific localisation to late Golgi cisternae as required for correct N‐glycosylation to occur, endogenous plant signals to both improve expression levels and suborganellar localisation were investigated.

### 2.3 Modifying HuGEs sub‐Golgi localisation using plant CTS domains

As type II membrane proteins, plant and human glycosylation enzymes both possess a single pass signal‐anchor sequence provided by a short N‐terminal CTS domain of about 40 to 70 amino acids which provides specific sub‐Golgi localisation and targets the enzyme's catalytic subunit to the Golgi lumen.^25^

**FIGURE 6:**
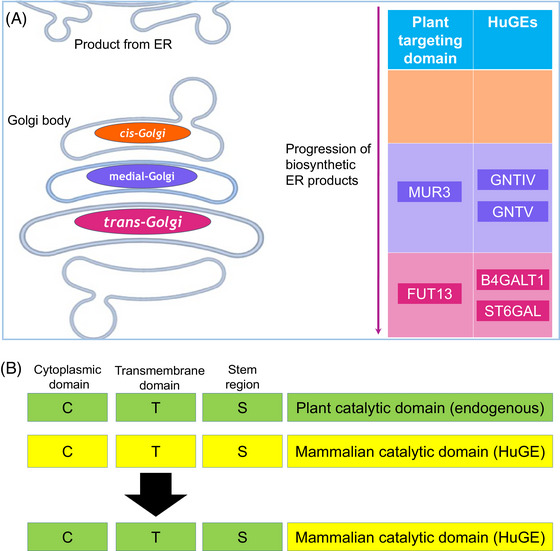
Schematic diagram of HuGE targeting approach. (A) HuGE targeting approach in relation to Golgi body structure. Cisternae localisation is colour‐coded (*cis* = orange, medial = purple, *trans* = magenta). The required localisation of the HuGEs and the plant CTS used for targeting are indicated: MUR3‐CTS to target GNTIV and GNTV to medial‐Golgi cisternae; FUT13‐CTS to target B4GALT1 and ST6GAL to *trans*‐Golgi cisternae. (B) Protein engineering approach for modified CTS‐HuGE constructs outlining domain swaps between mammalian and plant CTS domains.

With the aim to achieve Golgi cisternae‐specific targeting and to use plant‐derived sequences, HuGEs were targeted *in planta* using two CTS domains from *Arabidopsis thaliana* resident glycosylation enzymes: a MUR3‐CTS from β‐1,2‐galactosyltransferase as the medial‐Golgi targeting domain and a FUT13‐CTS from α‐1,4‐fucosyltransferase for *trans*‐Golgi targeting (Figure 6A).^25^ The mammalian CTS domains were located and replaced with the Arabidopsis CTS domains to create the following fusion proteins: MUR3‐GNTIV, MUR3‐GNTV, FUT13‐B4GALT1 and FUT13‐ST6GAL (Figure 6B).

**FIGURE 7:**
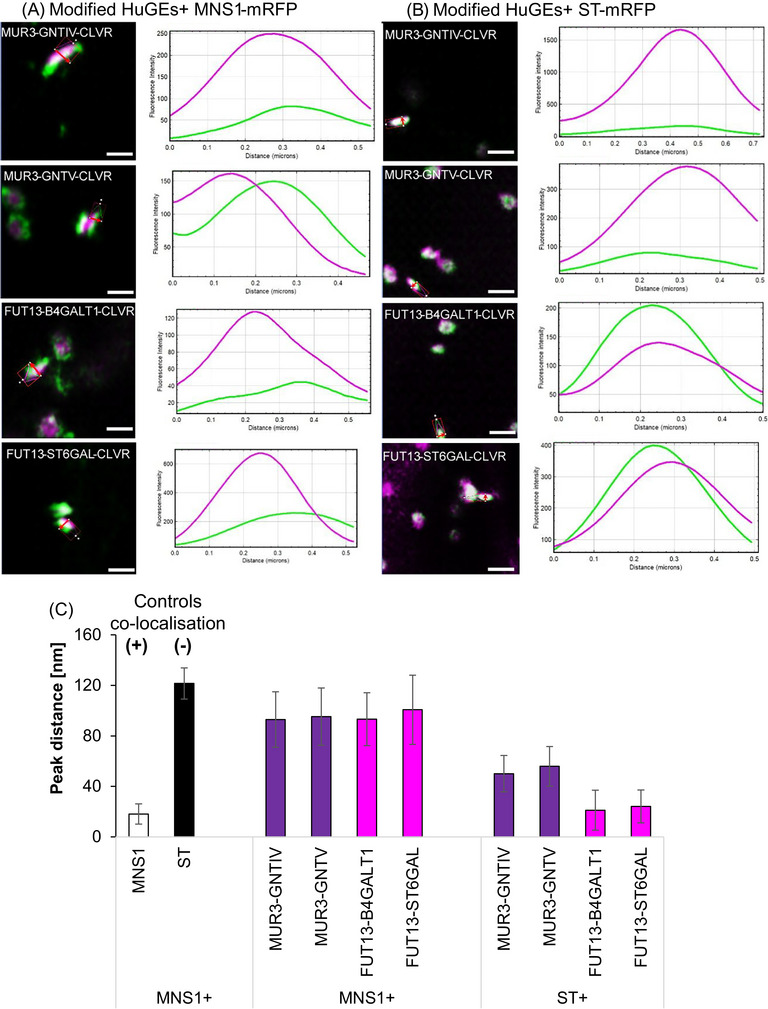
Co‐localisation line profile analysis for modified HuGEs. (A) Example images and analysis for distances between peak fluorescent intensities for MUR3‐GNTIV‐CLVR, MUR3‐GNTV‐CLVR, FUT13‐B4GALT1‐CLVR, FUT13‐ST6GAL‐CLVR (green), with *cis*‐Golgi marker MNS1‐mRFP or (B) medial/*trans*‐Golgi body marker ST‐mRFP, respectively (magenta). (C) Peak distance analysis graphs showing controls for co‐localisation in the same Golgi cisternae (MNS1‐eGFP+MNS1‐mRFP, white bar) and for localisation in different Golgi cisternae (MNS1‐eGFP+ST‐mRFP, black bar), as well as comparison of the four HuGEs (medial HuGEs in purple and *trans*‐Golgi HuGEs in magenta) with the respective markers MNS1‐mRFP and ST‐mRFP. Representative images are shown. Results shown from *n* = 4 biological replicas and >40 technical repeats for each combination. Size bars = 1 µm. Single channel images for Figure 7 are shown in Supplementary Figure S1.

The modified HuGE constructs were co‐expressed with both the *cis‐*Golgi marker MNS1 and the medial/*trans‐*Golgi marker ST in order to assess their suborganellar localisation (Figure 7; Supplementary Figure S1). Overall localisation was altered for HuGEs possessing plant CTS domains (Figure 7B). MUR3‐GNTIV did not co‐localise with MNS1 (maximum peak intensity distance 93.0 ± 21.9 nm) but showed a closer localisation to late Golgi marker ST‐mRFP (50.1 ± 14.4 nm). MUR3‐GNTV also did not co‐localise with MNS1 (95.3 ± 22.7 nm) but showed strong co‐localisation with the late Golgi cisternae (ST‐mRFP; 55.9 ± 15.7 nm). This indicated that the MUR3‐HuGEs were targeted to late Golgi cisternae.

*Trans*‐Golgi HuGEs FUT13‐B4GALT1 and FUT13‐ST6GAL also showed targeting to late Golgi cisternae. FUT13‐B4GALT1 did not co‐localise with MNS1 (93.2 ± 21.0 nm) but overlapped strongly with ST‐mRFP (21.1 ± 15.8 nm). FUT13‐ST6GAL showed little co‐localisation with MNS1 (100.7 ± 27.4 nm) but showed close localisation to ST‐mRFP (24.1 ± 13.0 nm). Overall MUR3‐HuGEs and FUT13‐HuGEs demonstrated more distant localisation from the *cis*‐Golgi marker MNS1‐mRFP (Figure 7B) than the native HuGEs (Figure 5). Interestingly, the FUT13‐HuGEs demonstrated a closer association with ST‐mRFP (Figure 7F) than the MUR3‐HuGEs.

This indicated that localisation to medial‐ and *trans*‐Golgi cisternae can be distinguished based on the distance between peak intensities of fluorescent protein fusions, determined by statistical line profile analysis. To follow this up we created a double marker construct intended to label two distinct cisternae of the plant Golgi simultaneously on a single construct. This construct contained the CTS of the medial marker MUR3 fused to a blue fluorescent protein (mTagBFP) followed by an intein sequence and P2A self‐cleaving peptide sequence^31^, ^32^ and the CTS of the *trans*‐Golgi marker FUT13 fused to mRFP. The usage of intein sequence and P2A allowed for expression of the two markers on the same plasmid but with MUR3‐CTS‐mTagBFP and FUT13‐CTS‐mRFP being cleaved into separate proteins, enabling equal stoichiometry of marker expression in the same cell while reducing the number of plasmids required for transformations. Self‐cleaving peptides such as P2A are short virus‐derived sequences which produce cleavage of polyproteins by a ribosomal skipping mechanism during translation between the last and second to last residues in the sequence.^33^ The intein sequence removes self‐cleaving peptide residues attached to the C‐terminus of MUR3‐CTS.^31^

**FIGURE 8:**
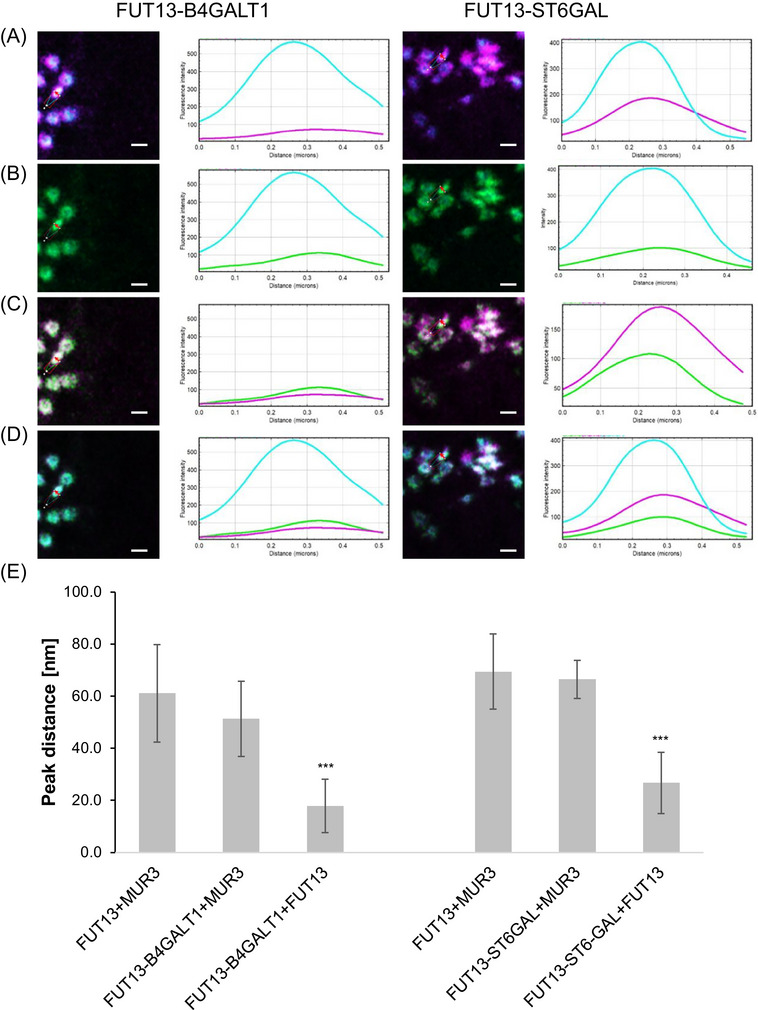
Co‐localisation line profile analysis for FUT13‐B4GALT1‐CLVR, FUT13‐ST6GAL‐CLVR with medial/*trans*‐Golgi marker construct. (A) Example images and line profile for the markers MUR3 (blue) and FUT13 (magenta), (B) FUT13‐B4GALT1‐CLVR or FUT13‐ST6GAL‐CLVR (green) with the medial‐Golgi marker MUR3 (blue), (C) FUT13‐B4GALT1‐CLVR or FUT13‐ST6GAL‐CLVR (green) with the *trans*‐Golgi marker FUT13 (magenta), (D) the overlay of all three proteins and the respective line profiles. Size bars = 1 µm. Single channel images for Figure 8 with higher magnification are shown in Supplementary Figure S2. (E) Peak distance analysis graphs showing the markers (MUR3+FUT13) and each marker with FUT13‐B4GALT1‐CLVR and FUT13‐ST6GAL‐CLVR, respectively. Significance was analysed by Kruskal–Wallis (***p < 0.001). Results shown from n = 3 biological replicas and 6 technical repeats.

The modified HuGEs FUT13‐B4GALT1‐CLVR and FUT13‐ST6GAL‐CLVR, respectively, were co‐expressed with the self‐cleaving peptide construct to allow for simultaneous comparison with both a medial‐ and a *trans*‐Golgi marker (Figure 8; Supplementary Figure S2). FUT13‐B4GALT1‐CLVR and FUT13‐ST6GAL‐CLVR showed a distance between maximum fluorescent intensities with the medial marker MUR3 of 51.3 ± 14.4 nm and 66.4 ± 7.3 nm, respectively (Figure 8B and E). These values aligned closely with the difference between the CTS domain markers MUR3 and FUT13 (61.1 ± 18.7 nm and 69.4 ± 14.5 nm, respectively; Figure 8A and E). In contrast both FUT13‐B4GALT1‐CLVR and FUT13‐ST6GAL‐CLVR co‐localised more closely with the *trans*‐Golgi marker FUT13 (17.81 ± 10.2 nm and 26.7 ± 11.8 nm, respectively; Figure 8C and E). Overall, this analysis showed that FUT13‐B4GALT1‐CLVR and FUT13‐ST6GAL‐CLVR are located in *trans*‐Golgi cisternae (Figure 8D and E).

To investigate if the targeting is solely CTS‐dependent or if the mammalian lumenal portions contribute to the suborganellar localisation, we analysed the CTS domains of MUR3 and FUT13 fused to fluorescent proteins (MUR3‐CTS‐eGFP and FUT13‐CTS‐eGFP; Supplementary Figure S3). FUT13‐CTS‐eGFP co‐localised with ST‐GFP (distance between maximum fluorescent intensities of 21.0 ± 14.6 nm) but not with MNS1‐eGFP (distance between maximum fluorescent intensities of 160.4 ± 24.8 nm; statistically significant with *p* < 0.001).

The distance between maximum fluorescent intensities between MUR3‐CTS‐eGFP and ST‐GFP was 80.9 ± 19.2 nm. The distance between maximum fluorescent intensities between MUR3‐CTS‐eGFP and MNS1‐eGFP was 145.5 ± 34.9 nm (statistically significant with *p* < 0.001). These data are of interest, as MUR3‐CTS seems to be less closely aligned with ST than FUT13‐CTS. This indicates not only a measurable separation between all three Golgi cisternae but also that ST is actually a *trans*‐Golgi marker^34^ rather than as often indicated a medial/*trans*‐Golgi marker.

Overall, the data showed that the Golgi cisternae localisation is indeed dependent on the CTS domain with no measurable impact from the enzymatic domain of the HuGEs.

Taken together, this suggested that CTS play a significant role in directing localisation of the HuGEs and show species specificity. Targeting of glycosylation enzymes to specific relevant sub‐Golgi cisternae was suggested to improve enzyme activity and to generate a greater yield of specific glycan structures; for example, GNTIV and GNTV produce a greater yield of bi‐ and tri‐ antennary glycans when expressed in the medial‐Golgi than when expressed in the *trans‐*Golgi.^5^, ^26^

Replacing the mammalian CTS with plant‐specific sequences has shown to be a valid approach for correct targeting and enhancing expression levels of mammalian HuGEs in plant systems. These successful protein engineering approaches are the first steps towards a plant‐based expression system for the production of therapeutic proteins of interest with modified N‐linked glycosylation. In addition, high‐resolution dynamic confocal microscopy and self‐cleaving peptide markers have been adapted as a robust and effective methodology for suborganellar protein localisation studies in Golgi body cisternae.

## 3 METHODS

### 3.1 Synthesis of human glycosylation enzyme sequences

The original human sequences for GNTIV, GNTV, B4GALT1 and ST6GAL were taken from the NCBI database. Human glycosylation enzymes were codon‐optimised for tobacco (Supplementary Data S4) and synthesised by TWIST Biosciences (San Francisco, USA, https://www.twistbioscience.com/).

### 3.2 Constructs for HuGEs with plant CTS replacements

The existing HuGE sequences previously obtained from the NCBI database were used as basis for CTS fusion design (Supplementary Data S4). The region upstream of the HuGE catalytic subunit was replaced with the appropriate plant‐specific CTS region (Table 1). For the medial‐Golgi HuGEs GNTIV and GNTV, MUR3 was used, and for the *trans*‐Golgi targeting B4GALT1 and ST6GAL, FUT13 was used (Supplementary Data S5). CTS‐replacement constructs were synthesised by TWIST Biosciences (San Francisco, USA, https://www.twistbioscience.com/).

**TABLE 1:** Putative CTS regions from *cis/*medial‐ and *trans*‐Golgi enzymes used as localisation strategies for novel HuGEs.^20^

Golgi enzyme: localisation & source	Cytoplasmic‐transmembrane‐stem region (CTS) with transmembrane domain underlined & bold	Total length of CTS region (aa)
MUR3: medial‐Golgi, *Arabidopsis thaliana* β‐1,2‐galactosyltransferase	MFPRVSMRRRSAEVSPTEPMEKGNGKNQTNR **ICLLVALSLFFWALLLYFHFVVL** GTSNIDKQLQLQPSYA	70 aa
FUT13: *trans‐*Golgi, *Arabidopsis thaliana* α‐1,4‐fucosyltransferase	MPMR **YLNAMAALLMMFFTLLILSFTGI** LEFPSASTSMEHSIDPEPKLSDSTS	52 aa

### 3.3 Vector preparation

Standard Gateway® cloning protocols were followed. Fusion constructs were first inserted into a pDONR^TM^221 Gateway Entry vector via BP Clonase II Enzyme Mix (ThermoFisher Scientific) according to the manufacturer's instructions and sequenced to confirm insertion and construct sequence. LR reactions were performed using an LR Clonase II Enzyme Mix (ThermoFisher) following manufacturer guidelines to generate the desired plant expression vector.^35^ The following Gateway destination vectors were used^35^: pB7RWG2 for C‐terminal mRFP fusions and pB7FWG2 for C‐terminal eGFP fusions. In the vector pB7FWG2 for C‐terminal eGFP fusions, the eGFP was replaced with a CLOVER green fluorescent protein (pB7FWG2:CLOVER).

Self‐cleaving peptide constructs^32^ containing the MUR3 (fused to mTagBFP) and FUT13 CTS domains separated by the self‐cleaving peptide P2A with intein sequence, were synthesised commercially (Twist) and supplied in an entry vector (for sequence see Supplementary Data S6). An LR reaction was carried out as described above with pB7RWG2 to add a C‐terminal mRFP fusions to the FUT13 CTS.

### 3.4 Preparation of competent Agrobacteria

The *Agrobacterium tumefaciens* strain GV3101^36^ was prepared as a large initial volume of 200 mL in LB (Lysogeny Broth media, with 25 µg/mL rifampicin) from a 28°C overnight culture. Bacteria were grown at 28°C in a shaking incubator until the culture reached an OD_600_ of 0.4. The initial volume of agrobacterium was then pelleted by centrifugation at 3500 rpm for 30 min, at 4°C. The pellet was resuspended in cold 1 M CaCl_2_ and then spun again at 3500 rpm for 10 min at 4°C. The pellet was resuspended in 15 mL 1 M CaCl_2_ and the bacterial solution was split into 400 µL aliquots snap‐frozen in liquid nitrogen and stored at −80°C.

### 3.5 Transformation of Agrobacteria

100 µL of competent Agrobacteria were combined with 400–600 ng of the expression plasmid of interest, and incubated on ice for 5 min. The mixture was then flash‐frozen either by liquid nitrogen immersion or by placement on metal shelving in the −80°C for 15 min. Agrobacteria were incubated in a 37°C water bath for 4 min. The mixture was rapidly transferred to a 15 mL Falcon tube with 1 mL LB and was then allowed to recover in a 28°C incubator with shaking in 1 mL fresh LB for 2–3 h. Agrobacteria were then spread onto an agar plate (25 µg/mL rifampicin, 50 µg/mL of gentamycin and either 50 µg/mL of kanamycin or spectinomycin dependent on the vector used), the plates were incubated at 28°C for 48 h, at which point colonies of agrobacterium could be observed. Transformed agrobacterium colonies were then transferred into liquid culture for use in agrobacterium‐mediated plant transformation.

### 3.6 Agrobacterium‐mediated transient protein expression in tobacco leaf epidermal cells

Agrobacterium‐mediated plant transformation was carried out as previously described.^36^, ^37^, ^38^ In brief, transformed agrobacterium liquid cultures were pelleted by centrifugation at 2200 × *g* at room temperature for 5 min. Infiltration buffer (5 mg/mL glucose, 50 mM MES, 2 mM Na_3_PO_4_· 12H_2_O and 0.1 mM acetosyringone) was made fresh from stocks and used to wash the pellet once, repellet by a repeat centrifugation step and then to resuspend the agrobacterium into a final solution. The bacterial suspension was diluted in the infiltration buffer to an OD_600_ = 0.1. The infiltration medium was injected into the abaxial leaf surface of 2‐week‐old mature tobacco plants (grown under a 16 h light/dark cycle) with a blunt 1 mL syringe, after first making a small puncture using a pipette tip. A permanent marker on the leaf surface was used to indicate the spread of the medium within the leaf tissue to ensure only infiltrated leaf surface is later used. Infiltrated plants were incubated at 23°C for 72 h before imaging.

### 3.7 Confocal microscopy

To image tobacco leaf epidermal cells, small leaf sections (25 mm^2^) were cut from infiltrated tobacco leaves and the abaxial surface images. Sections were mounted in water for confocal microscopy. All confocal microscopy was performed using a Zeiss LSM 880 with Airyscan detector. Images were collected with a Zeiss PlanApo 100x/1.46 NA oil immersion. 512 × 512 images were collected in 8‐bit with 2‐line averaging and excitation at 488 nm (eGFP) and 561 (mRFP), and emission at 495–550 nm and 570–615 nm, respectively.

### 3.8 Line profile analysis

**FIGURE 9:**
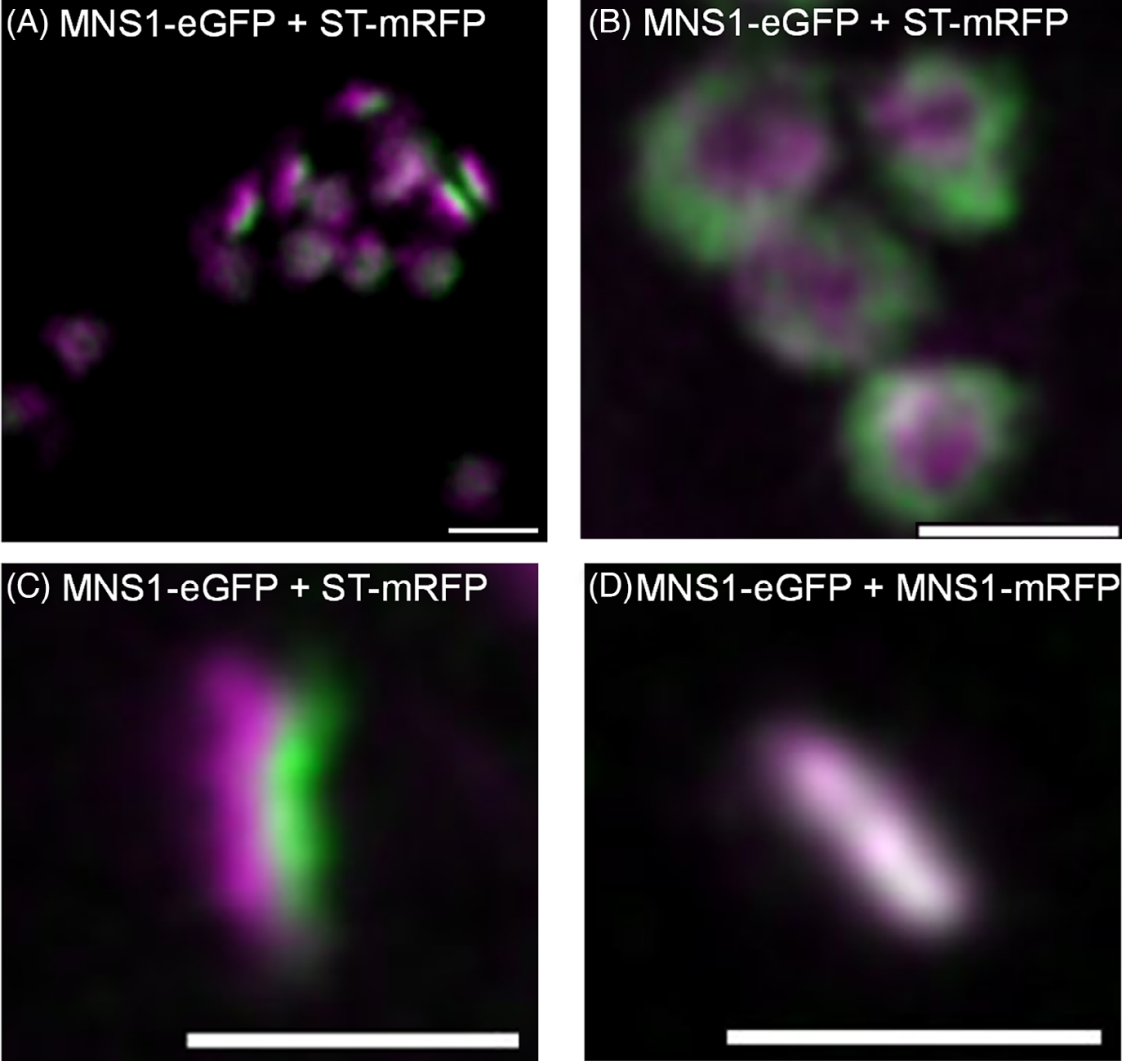
Microscopy images representative of Golgi bodies. (A) Wide view of offset between a *cis‐*Golgi marker (MNS1‐eGFP in green) and late Golgi marker (ST‐mRFP in magenta; (B) top‐down view of MNS1‐eGFP in green and ST‐mRFP in magenta; (C) Golgi body side profile suitable for analysis showing the MNS1‐eGFP in green and ST‐mRFP in magenta; (D) side profile suitable for analysis of individual Golgi body with co‐localisation of the *cis‐*Golgi marker MNS1 in two separate colours (MNS1‐eGFP in green and MNS1‐mRFP in magenta). Size bars = 1 µm.

Co‐localisation analysis for constructs and markers in the plant Golgi body was performed using the line profile utility in Zeiss ZEN blue software (Version 3.5.093.00000).^27^ The highly dynamic nature of imaging plant Golgi bodies necessitated use of long time series to collect a sufficient number of suitable side‐profile images while Golgi rotate a prerequisite to co‐localisation analysis (Figure 9A). It is not possible to visualise cisternae separation in flat or top‐down views of the Golgi (Figure 9B) due to overlapping cisternae resulting in overlapping fluorescent signal, with individual cisternae only distinct in side profile (Figure 9C). Individual frames of time series were used to locate suitable side‐profile Golgi (Figure 9C and D) to visualise cisternae and determine degree of co‐localisation.

**FIGURE 10:**
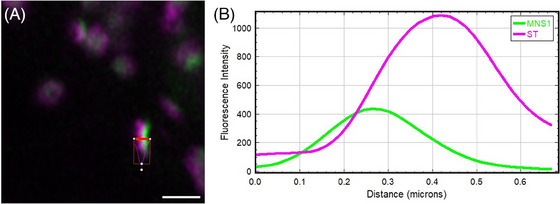
(A) Example profile view confocal image and (B) output of a line intensity analysis from ZEN blue for microscopy data for ST‐mRFP (magenta) and MNS1‐eGFP (green).

With suitable side profile Golgi obtained, ZEN blue software was used to draw a line perpendicular to the selected Golgi body (Figure 9) in order to generate a line intensity profile (Figure 10) of the two channels.

**FIGURE 11:**
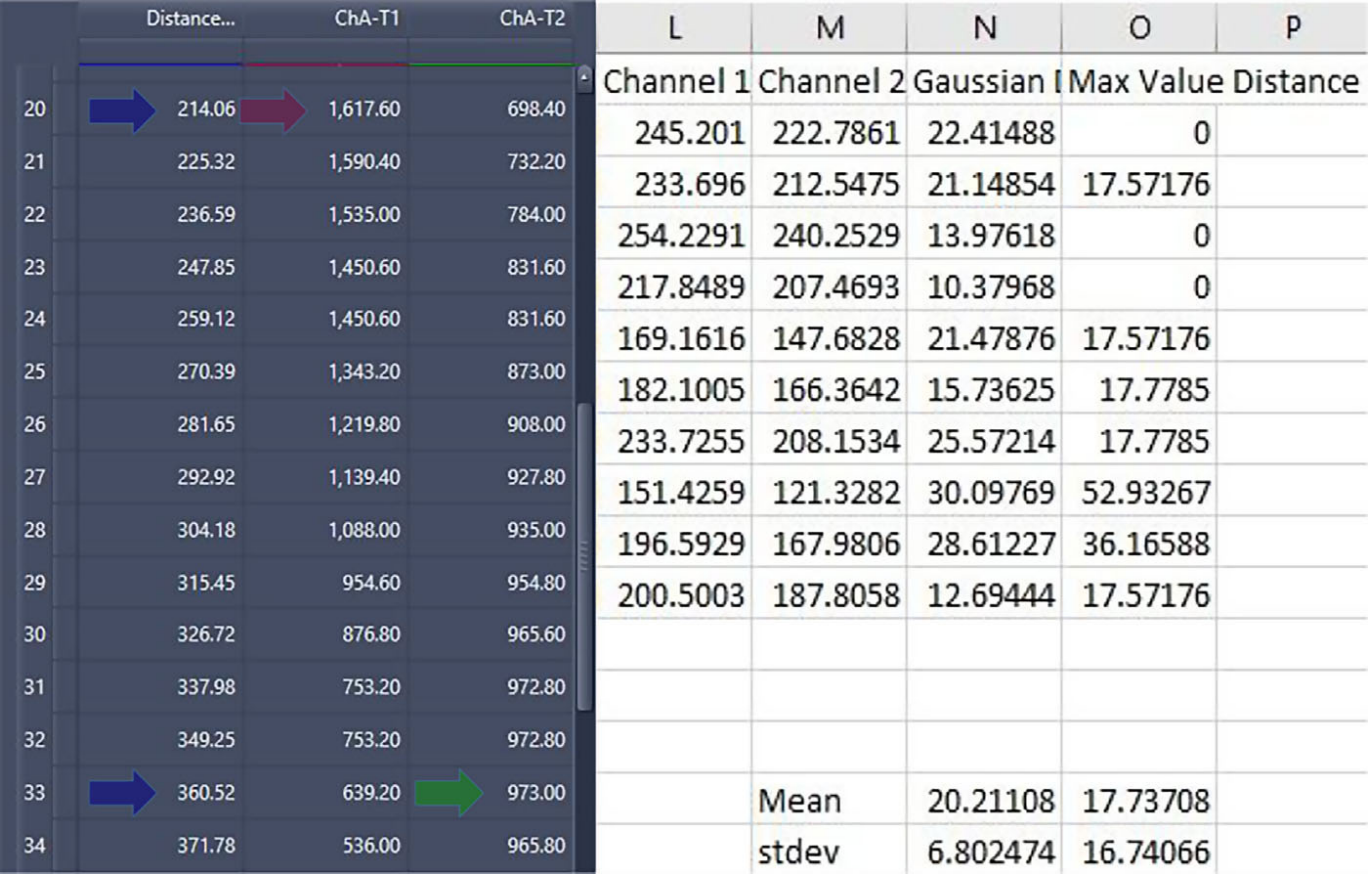
Data output for line profiles. Example data output of line profile analysis in Zen is shown on the left hand side. Maximum fluorescence intensity of channel 1 (red arrow) and maximum of channel 2 (green arrow) is indicated, with the distance separating the two peaks along the line intensity profile (blue arrows). An example of a data subset is shown on the right hand side. Each row provides a single Golgi body line profile analysis from one combination showing maximum intensities and the distance between them, processed by use of a Python script.

The distance between the maximum peak intensities of each channel was collected across the line profile for each Golgi body analysed (Figure 11), with the data being used to determine the maximum peak intensity of each respective channel (red and green arrows), and the distance value between them in nm (blue arrows). Average and standard deviation were calculated for each protein combination.

This process was automated through use of a Python script (Figure 9; Supplementary Data S7 with code for Python version 3.9) to iterate through multiple .csv files, obtain distance between peaks and fit to a Gaussian distribution to improve precision of results collected, as ZEN only formats distance measurements by pixel, leading to an irregular scale that lacks small increments. This delivers peak intensity of each channel, as either the modified Gaussian‐fitted data or the unmodified max distance data delivered by Zen, to compare for any errors, then used to produce averages and standard deviations across collected line intensity profiles.

## AUTHOR CONTRIBUTIONS

VK conceived of the study together with JS, RS and SB. Experiments and data analysis were performed by AMG, VK and JS. VK wrote the manuscript with contributions from all the authors listed. VK agrees to serve as the author responsible for contact and ensures communication.

## Supporting information

Supporting information
