# Supplementary material for: Suborganellar resolution imaging for the localisation of human glycosylation enzymes in tobacco Golgi bodies
Source: J Microsc. 2024 Apr 30;297(3):258–73. doi: 10.1111/jmi.13311 (PMC11808412; doi:10.1111/jmi.13311)
Supplement: Supplementary file 1 — Supporting information [file JMI-297-258-s001.docx]

**Supplementary Data**

Supplementary Figure S1

Supplementary Figure S1: Representative single channel images for modified HuGEs from Figure 7.

A) Representative images (single channels green and red as well as merged channel) for MUR3-GNTIV-CLVR, MUR3-GNTV-CLVR, FUT13-B4GALT1-CLVR, FUT13-ST6GAL-CLVR (green), with *cis*-Golgi marker MNS1-mRFP or B) medial/*trans*-Golgi body marker ST-mRFP, respectively (magenta). Size bars = 1µm.

Supplementary Figure S2

Supplementary Figure S2: Representative and high-resolution images for FUT13-B4GALT1-CLVR, FUT13-ST6GAL-CLVR with medial/*trans*-Golgi marker construct from Figure 8.

A) Example images for FUT13-B4GALT1-CLVR or FUT13-ST6GAL-CLVR (green), B) the marker FUT13 (magenta), C) the medial-Golgi marker MUR3 (blue). D) shows the overlay of all three proteins.

Supplementary Figure S3

Supplementary Figure S3: Peak distance analysis for constructs with CTS domain only (MUR3-CTS and FUT13-CTS).

Peak distance analysis graphs showing co-localisation analysis for MUR3-CTS-mRFP and FUT13-CTS-mRFP, respectively, with the markers MNS1-eGFP (*cis*-Golgi cisternae, orange) and ST-GFP (medial/*trans*-Golgi cisternae, pink). Results shown from n = 3 biological replicas and >28 technical repeats for each combination. Significance was analysed by Kruskal-Wallis (***p< 0.001).

Supplementary Data S4. Native HuGE enzyme sequence

**GNTIVa**

>sp|Q9UM21|MGT4A_HUMAN Alpha-1,3-mannosyl-glycoprotein 4-beta-N-acetylglucosaminyltransferase A OS=Homo sapiens OX=9606 GN=MGAT4A PE=1 SV=1

MRLRNGTVATALAFITSFLTLSWYTTWQNGKEKLIAYQREFLALKERLRIAEHRISQRSSELNTIVQQFKRVGAETNGSKDALNKFSDNTLKLLKELTSKKSLQVPSIYYHLPHLLKNEGSLQPAVQIGNGRTGVSIVMGIPTVKREVKSYLIETLHSLIDNLYPEEKLDCVIVVFIGETDIDYVHGVVANLEKEFSKEISSGLVEVISPPESYYPDLTNLKETFGDSKERVRWRTKQNLDYCFLMMYAQEKGIYYIQLEDDIIVKQNYFNTIKNFALQLSSEEWMILEFSQLGFIGKMFQAPDLTLIVEFIFMFYKEKPIDWLLDHILWVKVCNPEKDAKHCDRQKANLRIRFRPSLFQHVGLHSSLSGKIQKLTDKDYMKPLLLKIHVNPPAEVSTSLKVYQGHTLEKTYMGEDFFWAITPIAGDYILFKFDKPVNVESYLFHSGNQEHPGDILLNTTVEVLPFKSEGLEISKETKDKRLEDGYFRIGKFENGVAEGMVDPSLNPISAFRLSVIQNSAVWAILNEIHIKKATN

**GNTV**

>sp|Q09328|MGT5A_HUMAN Alpha-1,6-mannosylglycoprotein 6-beta-N-acetylglucosaminyltransferase A OS=Homo sapiens OX=9606 GN=MGAT5 PE=1 SV=1

MALFTPWKLSSQKLGFFLVTFGFIWGMMLLHFTIQQRTQPESSSMLREQILDLSKRYIKALAEENRNVVDGPYAGVMTAYDLKKTLAVLLDNILQRIGKLESKVDNLVVNGTGTNSTNSTTAVPSLVALEKINVADIINGAQEKCVLPPMDGYPHCEGKIKWMKDMWRSDPCYADYGVDGSTCSFFIYLSEVENWCPHLPWRAKNPYEEADHNSLAEIRTDFNILYSMMKKHEEFRWMRLRIRRMADAWIQAIKSLAEKQNLEKRKRKKVLVHLGLLTKESGFKIAETAFSGGPLGELVQWSDLITSLYLLGHDIRISASLAELKEIMKKVVGNRSGCPTVGDRIVELIYIDIVGLAQFKKTLGPSWVHYQCMLRVLDSFGTEPEFNHANYAQSKGHKTPWGKWNLNPQQFYTMFPHTPDNSFLGFVVEQHLNSSDIHHINEIKRQNQSLVYGKDSFWKNKKIYLDIIHTYMEVHATVYGSSTKNIPSYVKNHGILSGRDLQFLLRETKLFVGLGFPYEGPAPLEAIANGCAFLNPKFNPPKSSKNTDFFIGKPTLRELTSQHPYAEVFIGRPHVWTVDLNNQEEVEDAVKAILNQKIEPYMPYEFTCEGMLQRINAFIEKQDFCHGQVMWPPLSALQVKLAEPGQSCKQVCQESQLICEPSFFQHLNKDKDMLKYKVTCQSSELAKDILVPSFDPKNKHCVFQGDLLLFSCAGAHPRHQRVCPCRDFIKGQVALCKDCL

##

## B4GALT1

>sp|P15291|B4GT1_HUMAN Beta-1,4-galactosyltransferase 1 OS=Homo sapiens OX=9606 GN=B4GALT1 PE=1 SV=5

MRLREPLLSGSAAMPGASLQRACRLLVAVCALHLGVTLVYYLAGRDLSRLPQLVGVSTPLQGGSNSAAAIGQSSGELRTGGARPPPPLGASSQPRPGGDSSPVVDSGPGPASNLTSVPVPHTTALSLPACPEESPLLVGPMLIEFNMPVDLELVAKQNPNVKMGGRYAPRDCVSPHKVAIIIPFRNRQEHLKYWLYYLHPVLQRQQLDYGIYVINQAGDTIFNRAKLLNVGFQEALKDYDYTCFVFSDVDLIPMNDHNAYRCFSQPRHISVAMDKFGFSLPYVQYFGGVSALSKQQFLTINGFPNNYWGWGGEDDDIFNRLVFRGMSISRPNAVVGRCRMIRHSRDKKNEPNPQRFDRIAHTKETMLSDGLNSLTYQVLDVQRYPLYTQITVDIGTPS

## ST6GAL1

>sp|P15907|SIAT1_HUMAN Beta-galactoside alpha-2,6-sialyltransferase 1 OS=Homo sapiens OX=9606 GN=ST6GAL1 PE=1 SV=1

MIHTNLKKKFSCCVLVFLLFAVICVWKEKKKGSYYDSFKLQTKEFQVLKSLGKLAMGSDSQSVSSSSTQDPHRGRQTLGSLRGLAKAKPEASFQVWNKDSSSKNLIPRLQKIWKNYLSMNKYKVSYKGPGPGIKFSAEALRCHLRDHVNVSMVEVTDFPFNTSEWEGYLPKESIRTKAGPWGRCAVVSSAGSLKSSQLGREIDDHDAVLRFNGAPTANFQQDVGTKTTIRLMNSQLVTTEKRFLKDSLYNEGILIVWDPSVYHSDIPKWYQNPDYNFFNNYKTYRKLHPNQPFYILKPQMPWELWDILQEISPEEIQPNPPSSGMLGIIIMMTLCDQVDIYEFLPSKRKTDVCYYYQKFFDSACTMGAYHPLLYEKNLVKHLNQGTDEDIYLLGKATLPGFRTIHC

Supplementary Data S5. Modified CTS-HUGE sequences

**MUR3-GNTIV**

MFPRVSMRRRSAEVSPTEPMEKGNGKNQTNRICLLVALSLFFWALLLYFHFVVLGTSNIDKQLQLQPSYATIVQQFKRVGAETNGSKDALNKFSDNTLKLLKELTSKKSLQVPSIYYHLPHLLKNEGSLQPAVQIGNGRTGVSIVMGIPTVKREVKSYLIETLHSLIDNLYPEEKLDCVIVVFIGETDIDYVHGVVANLEKEFSKEISSGLVEVISPPESYYPDLTNLKETFGDSKERVRWRTKQNLDYCFLMMYAQEKGIYYIQLEDDIIVKQNYFNTIKNFALQLSSEEWMILEFSQLGFIGKMFQAPDLTLIVEFIFMFYKEKPIDWLLDHILWVKVCNPEKDAKHCDRQKANLRIRFRPSLFQHVGLHSSLSGKIQKLTDKDYMKPLLLKIHVNPPAEVSTSLKVYQGHTLEKTYMGEDFFWAITPIAGDYILFKFDKPVNVESYLFHSGNQEHPGDILLNTTVEVLPFKSEGLEISKETKDKRLEDGYFRIGKFENGVAEGMVDPSLNPISAFRLSVIQNSAVWAILNEIHIKKATN

**MUR3-GNTV**

MFPRVSMRRRSAEVSPTEPMEKGNGKNQTNRICLLVALSLFFWALLLYFHFVVLGTSNIDKQLQLQPSYATNSTNSTTAVPSLVALEKINVADIINGAQEKCVLPPMDGYPHCEGKIKWMKDMWRSDPCYADYGVDGSTCSFFIYLSEVENWCPHLPWRAKNPYEEADHNSLAEIRTDFNILYSMMKKHEEFRWMRLRIRRMADAWIQAIKSLAEKQNLEKRKRKKVLVHLGLLTKESGFKIAETAFSGGPLGELVQWSDLITSLYLLGHDIRISASLAELKEIMKKVVGNRSGCPTVGDRIVELIYIDIVGLAQFKKTLGPSWVHYQCMLRVLDSFGTEPEFNHANYAQSKGHKTPWGKWNLNPQQFYTMFPHTPDNSFLGFVVEQHLNSSDIHHINEIKRQNQSLVYGKVDSFWKNKKIYLDIIHTYMEVHATVYGSSTKNIPSYVKNHGILSGRDLQFLLRETKLFVGLGFPYEGPAPLEAIANGCAFLNPKFNPPKSSKNTDFFIGKPTLRELTSQHPYAEVFIGRPHVWTVDLNNQEEVEDAVKAILNQKIEPYMPYEFTCEGMLQRINAFIEKQDFCHGQVMWPPLSALQVKLAEPGQSCKQVCQESQLICEPSFFQHLNKDKDMLKYKVTCQSSELAKDILVPSFDPKNKHCVFQGDLLLFSCAGAHPRHQRVCPCRDFIKGQVALCKDCL

**FUT13-B4GALT1**

MPMRYLNAMAALLMMFFTLLILSFTGILEFPSASTSMEHSIDPEPKLSDSTSLPACPEESPLLVGPMLIEFNMPVDLELVAKQNPNVKMGGRYAPRDCVSPHKVAIIIPFRNRQEHLKYWLYYLHPVLQRQQLDYGIYVINQAGDTIFNRAKLLNVGFQEALKDYDYTCFVFSDVDLIPMNDHNAYRCFSQPRHISVAMDKFGFSLPYVQYFGGVSALSKQQFLTINGFPNNYWGWGGEDDDIFNRLVFRGMSISRPNAVVGRCRMIRHSRDKKNEPNPQRFDRIAHTKETMLSDGLNSLTYQVLDVQRYPLYTQITVDIGTPS

**FUT13-ST6GAL**

MPMRYLNAMAALLMMFFTLLILSFTGILEFPSASTSMEHSIDPEPKLSDSTSASFQVWNKDSSSKNLIPRLQKIWKNYLSMNKYKVSYKGPGPGIKFSAEALRCHLRDHVNVSMVEVTDFPFNTSEWEGYLPKESIRTKAGPWGRCAVVSSAGSLKSSQLGREIDDHDAVLRFNGAPTANFQQDVGTKTTIRLMNSQLVTTEKRFLKDSLYNEGILIVWDPSVYHSDIPKWYQNPDYNFFNNYKTYRKLHPNQPFYILKPQMPWELWDILQEISPEEIQPNPPSSGMLGIIIMMTLCDQVDIYEFLPSKRKTDVCYYYQKFF

Supplementary Data S6. Synthesised and codon-optimised medial/*trans* Golgi marker with self-cleaving peptide

MUR3-linker-mTagBFP-Intein-linker-P2A-FUT13/CTS-marker

ATGTTCCCCAGAGTCAGTATGCGAAGGAGAAGTGCAGAGGTCAGCCCCACTGAACCCATGGAAAAGGGCAACGGTAAGAACCAAACAAACCGCATTTGCCTCTTGGTCGCGCTGAGCTTGTTCTTTTGGGCGCTTCTATTATATTTTCACTTCGTAGTACTTGGTACCAGCAATATAGACAAGCAATTACAGCTTCAGCCATCCTACGCATGCAGTTGCGGGTCCGGAAGTAGGATGGTAAGTAAAGGAGAGGAATTAATCAAAGAAAATATGCATATGAAGCTTTATATGGAAGGAACAGTTGATAATCACCATTTTAAATGTACTTCTGAAGGGGAAGGGAAACCTTATGAGGGTACTCAAACAATGCGCATAAAAGTTGTTGAAGGTGGTCCATTACCATTTGCGTTTGATATTCTCGCCACAAGTTTTCTATATGGGTCTAAAACATTTATTAATCATACACAGGGAATCCCAGATTTCTTTAAACAGTCATTTCCAGAAGGTTTTACTTGGGAACGGGTTACAACTTATGAGGATGGTGGTGTTTTAACAGCCACACAAGATACTAGTTTGCAAGACGGGTGTTTAATTTATAACGTTAAAATTCGGGGAGTAAATTTTACCTCAAATGGACCAGTAATGCAGAAGAAGACTTTGGGGTGGGAAGCTTTTACTGAAACATTGTATCCGGCAGATGGTGGGCTCGAGGGACGTAATGATATGGCATTGAAATTGGTCGGTGGTAGTCACCTTATTGCCAATGCTAAAACAACGTACAGGAGCAAGAAGCCAGCTAAGAATTTGAAAATGCCCGGTGTTTATTACGTGGATTATAGGCTTGAGAGGATTAAAGAAGCAAATAATGAAACATATGTTGAACAACATGAAGTCGCTGTCGCTAGGTATTGTGATCTTCCATCCAAGTTGGGTCATAAATTAAACTGCCTGTCTTTTGGTACAGAAATCCTCACTGTGGAGTACGGCCCTTTGCCAATAGGGAAAATCGTTTCTGAGGAAATTAATTGTTCCGTGTACTCTGTAGACCCCGAAGGCAGGGTCTATACGCAGGCTATAGCACAATGGCACGACCGAGGGGAGCAAGAAGTCCTGGAGTACGAACTCGAGGACGGCTCAGTTATCCGTGCAACGTCTGACCATAGATTCTTGACAACTGACTATCAGTTGTTAGCTATAGAGGAAATATTTGCACGCCAACTTGACCTGCTGACCCTCGAGAACATAAAACAGACTGAGGAAGCCCTCGACAATCACAGACTGCCTTTCCCACTACTCGACGCGGGGACAATTAAAATGGTCAAGGTTATTGGTCGGCGTTCTCTCGGAGTACAGAGGATTTTCGACATTGGTTTACCCCAAGACCATAACTTCTTGTTGGCGAATGGCGCAATAGCAGCGGCCTGCAGTTGCGGCTCAGGCAGTCGTGGCTCTGGCGCAACTAACTTTTCCCTTCTCAAGCAAGCAGGTGATGTAGAAGAAAACCCAGGCCCGATGCCGATGCGATATTTGAATGCCATGGCTGCCCTGCTTATGATGTTCTTCACGCTTTTAATTTTAAGTTTTACTGGAATACTTGAGTTTCCTTCCGCAAGCACCTCTATGGAACACAGTATTGATCCAGAGCCCAAGTTAAGCGACAGCACTAGTC

Supplementary Data S7. Python script for peak intensity distance

import numpy as np

from glob import glob

import pandas as pd

from scipy.optimize import curve_fit

import matplotlib.pyplot as plt

# How to use:

# Put all csv files in Data Folder

# Run main.py

# Output Figures will appear in Output Figures folder

# Define gaussian equation

def gauss(x,a,b,c):

return a*np.exp(-((x-b)**2) / (2*c**2))

# Get all csv file names in Data folder

pattern = "Data/*.csv"

file_name_list = glob(pattern)

# Alphabetise (glob reads files in a random order)

file_name_list = np.sort(file_name_list)

print(f"Number of Files Found: {len(file_name_list)}\n")

# Empty list to store distances

gauss_distances_list = []

max_value_distances_list = []

channel_1_max_list = []

channel_2_max_list = []

# Loop through file names

for i, file in enumerate(file_name_list):

# Load data

data = np.genfromtxt(file, delimiter=',', skip_header=3)

# Get channel data (unnecessary, but i'm making an effort to change variables into something less abstract)

channel_1 = data[:,1]

channel_2 = data[:,2]

distances = data[:,0]

# The fit doesn't have to have the same number of datapoints, it looks smoother with 1000 points

x_values = np.linspace(0, np.max(distances), 1000)

# Fit Channel 1 Data to Gaussian

# Guesses 1

height = np.max(channel_1)

position = distances[int(np.argmax(channel_1))]

stdev = 100

guesses = [height, position, stdev]

# Get residuals and create fit function

fit_values_1, _ = curve_fit(gauss, xdata=distances, ydata=channel_1, p0=guesses)

y_fit = gauss(x_values, fit_values_1[0], fit_values_1[1], fit_values_1[2])

# Plot data as scatter and fit as line

plt.figure(i, figsize=(7,5))

plt.scatter(distances, channel_1, color="#ca0cf5", label="Channel 1 Data")

plt.plot(x_values, y_fit, color="#ca0cf5", label="Channel 1 Fit")

# Fit Channel 2 Data to Gaussian

# Guesses 2

height = np.max(channel_2)

position = distances[int(np.argmax(channel_2))]

stdev = 100

guesses = [height, position, stdev]

# Get residuals and create fit function

fit_values_2, _ = curve_fit(gauss, xdata=distances, ydata=channel_2, p0=guesses)

y_fit = gauss(x_values, fit_values_2[0], fit_values_2[1], fit_values_2[2])

# Plot data as scatter and fit as line

plt.scatter(distances, channel_2, color="#2bff00", label="Channel 2 Data")

plt.plot(x_values, y_fit, color="#2bff00", label="Channel 2 Fit")

# Graph formatting

plt.legend()

plt.xlabel("Distance (nm)")

plt.ylabel("Intensity")

plt.title(file[5:-4])

plt.tight_layout()

fig_title = f"./Output_Figures/{file[5:-4]}.png"

plt.savefig(fig_title)

# Calculate distance between peak locations using gaussian distribution

gaus_separation = abs(fit_values_1[1] - fit_values_2[1])

gauss_distances_list.append(gaus_separation)

# Calculate distance between peak locations using maximum value

max_value_sep = abs(distances[np.argmax(channel_1)] - distances[np.argmax(channel_2)])

max_value_distances_list.append(max_value_sep)

channel_1_max_list.append(fit_values_1[1])

channel_2_max_list.append(fit_values_2[1])

print("File Name:")

print(file[5:-4])

print(f"Distance: {np.round(gaus_separation, 2)} nm\n")

# Store it in a CSV that excel can open

dictionary = {"File Name": file_name_list,

"Channel 1 Peak Location": channel_1_max_list,

"Channel 2 Peak Location": channel_2_max_list,

"Gaussian Distance (nm)": gauss_distances_list,

"Max Value Distance (nm)": max_value_distances_list}

df = pd.DataFrame(dictionary)

df.to_csv("Analysis.csv")
